# Etiology-specific optimal timing of surgical intervention for intracranial hemorrhage: a systematic review and network meta-analysis

**DOI:** 10.1097/JS9.0000000000004591

**Published:** 2025-12-19

**Authors:** Nada Mostafa Al-Dardery, Dina Essam Abo-Elnour, Sadil Mohammad Bani Khaled, Alyaa Khaled Madeeh, Shahd Alqato, Suhel.F. Batarseh, Mariam A. Abusalah, Amr Diaaeldin Sayed Mahmoud, Wesal Nasr Mahmoud, Abdulrhman M Khaity

**Affiliations:** a Faculty of Medicine, Fayoum University, Fayoum, Egypt; b Faculty of Medicine, Zagazig University, Zagazig, Egypt; c Faculty of Medicine, The University of Jordan, Amman, Jordan; d Internal Medicine Department, Arab Medical Center, Amman, Jordan; e Faculty of Medicine, Jordan University of Science and Technology, Irbid, Jordan; f Faculry of Medicine, Al Quds University, Al Azhar branch, Gaza, Palestine; g Faculty of Medicine, Elrazi University, Khartoum, Sudan

**Keywords:** intracranial hemorrhage, network meta-analysis, neurosurgery, surgical timing

## Abstract

**Background:**

Intracranial hemorrhages (ICrH), including intracerebral hemorrhage (ICH) and aneurysmal subarachnoid hemorrhage (aSAH), are life-threatening conditions leading to high mortality and long-term disability. A persistent challenge in clinical practice is identifying the most advantageous timing for surgical intervention, as existing literature reveals conflicting outcomes regarding survival, neurological recovery, and complication rates. The literature remains heterogeneous despite extensive research, presenting conflicting evidence regarding survival benefits across various surgical timeframes, from ultra-early interventions (<6 hours) to delayed interventions (>7 days). This study aimed to determine the optimal timing for surgery in intracranial bleeding by comparing outcomes across timeframes to guide evidence-based clinical decisions.

**Methods:**

A systematic search of PubMed, Scopus, Web of Science, and Cochrane Library was conducted for studies published from 2000 to 2025. Twenty-eight observational studies, including 5919 patients, were included. Data were analyzed using random-effects models in R software, with subgroup analyses based on the time windows and type of hemorrhage.

**Results:**

Surgery performed within 24 hours significantly reduced the odds of poor GOS scores (OR: 0.53, 95% CI: 0.31–0.92) and rebleeding (OR: 0.55, 95% CI: 0.37–0.80). The network meta-analysis indicated that surgery within 48 hours is the most effective timing for reducing mortality (*P*-score = 0.99). However, pairwise comparisons revealed non-significant effects (OR = 0.94, 95% CI: 0.51–1.72), underscoring the necessity for etiology-specific interpretation. Early intervention (≤72 hours) was most beneficial in aSAH, whereas ultra-early surgery (<6 hours) in traumatic ICH showed higher mortality. Surgery delayed beyond 7 days resulted in worse functional recovery.

**Conclusions:**

Early surgical intervention, particularly within 24–48 hours, improves outcomes in many cases of ICH. However, optimal timing varies with hemorrhage type. The findings resolve previous discrepancies by illustrating that treatment effects are contingent upon both the timeframe and the underlying pathology. Personalized timing strategies are essential, and further high-quality randomized trials are needed to refine clinical guidelines.

## Background

Intracranial hemorrhage (ICrH) can be categorized into four main types: epidural, subdural, subarachnoid (SAH), and intracerebral hemorrhage (ICH)[1]. ICH is defined as a hematoma within the brain parenchyma resulting from the rupture of a blood vessel[2]. Although ICHs are less common than ischemic strokes, they are associated with high mortality and disability rates[3]. ICH is associated with various complications, including hematoma expansion, hydrocephalus, increased intracranial pressure, elevated blood pressure, and seizures[4]. SAH is a subtype of stroke that is caused by a ruptured aneurysm in about 85% of cases[5]. The outcome after aneurysmal subarachnoid hemorrhages (aSAH) depends on multiple factors, such as the severity of the condition, rebleeding, timing, and success of aneurysmal exclusion from circulation[6]. It is worth mentioning that although cerebral aneurysms are usually associated with SAH, they can cause ICH, complicating the course of the disease[7].

The timing of surgical intervention in ICrH varies considerably across studies, often classified into broad categories such as ultra-early, early, and delayed surgery^[8–10]^. However, there is no universally accepted definition for these categories, contributing to inconsistencies in reported outcomes^[8–10]^. In most studies, ultra-early surgery refers to intervention within the first 6 hours after symptom onset or diagnosis, early surgery between 6 and 24 hours, and delayed surgery after 24 hours^[8–10]^. Some reports further subdivide delayed surgery (e.g., > 48 hours, > 7 days), particularly in cases of aSAH^[11,12]^.

While some clinical studies have shown benefits for early evacuation, others have reported no benefit or worse situations. For example, while removing the hematoma during the ultra-early time (≤6 hours) reduces the secondary brain damage due to the decreased compression time, complications such as secondary bleeding may occur, which is the reason why some clinicians prefer to do the surgery within 7–24 hours of bleeding^[13–15]^. Literature is also not consistent regarding the effect of surgery timing on the outcomes in patients with aneurysmal SAH. For example, Muangman *et al* found that, generally, there was no difference in major complications between the ultra-early (within 24 hours) and early surgical (>24 hours) groups[16]. A second study by Dellaretti *et al* showed that patients who underwent late surgery (>10 days) had better outcomes than those who underwent intermediate surgery (3–10 days), with comparable outcomes to those who underwent early surgery (<48 hours) or intermediate surgery (3–10 days)[17]. These findings reveal the presence of controversy on the optimal surgical timing for patients with intracranial hematomas.

Although several studies have attempted to clarify optimal surgical timing in ICrH, most have approached the condition as a single, uniform entity or relied on simplified early-versus-delayed comparisons^[10,12,18]^. Such approaches overlook the distinct biological mechanisms and clinical trajectories associated with hypertensive ICH, traumatic ICH, and aneurysmal SAH, limiting the applicability of their conclusions^[10,12,18]^. Additionally, earlier reviews often reclassified timing definitions into standardized categories, which may obscure meaningful differences between studies^[10,12,18]^. To advance the field, our work introduces an etiology-centered framework combined with a network meta-analyses (NMA) model capable of comparing multiple timing intervals across heterogeneous datasets. By retaining each study’s original timing definitions and evaluating timing effects separately for each hemorrhage subtype, this study provides a more precise and clinically aligned assessment than previously available analyses.

## Methods

This systematic review and NMA were performed according to the Preferred Reporting Items for Systematic Reviews and Network Meta-Analyses (PRISMA-NMA)[19] and the AMSTAR Guidelines.^[20,21]^ Every step was done according to the Cochrane Handbook of Systematic Reviews and Meta-analysis of Interventions.[22] The study protocol was registered in PROSPERO. This study maintains methodological transparency by conforming to accepted reporting standards, including the TITAN guideline for robust scientific communication[23].

### Search strategy and selection process

A comprehensive search was conducted through PubMed, Scopus, Cochrane Library, and Web of Science from January 2000 until February 2025. Publications in English were systematically reviewed. During the search process, a combination of medical subject headings and keywords was employed as follows: “subarachnoid hemorrhage,” “intracerebral hemorrhage,” “intracranial aneurysm,” “craniotomy,” “surgical,” “operative,” “microsurgical,” “time factors,” “early,” “delay,” “timing.”

**HIGHLIGHTS**

- Surgery within 24–48 hours offers the best outcomes for many intracranial hemorrhage patients, lowering death rates and improving recovery in certain types.
- The best timing depends on the type of hemorrhage, as early surgery is not always helpful – especially in aneurysmal or traumatic cases.
- Delaying surgery beyond 72 hours is linked to worse survival and limited benefit for other complications.
- The study supports a tailored approach using hemorrhage type, imaging, and future research to guide treatment timing.

Supplemental Digital Content, Table 1, available at: http://links.lww.com/JS9/G454 presents a comprehensive search strategy for each database. The reference lists of the selected publications were examined manually, and duplicate records were removed using EndNote software. Eligibility was determined via a two-step screening process, including an initial review of titles and abstracts and a comprehensive evaluation of full texts. Two reviewers conducted independent assessments of each study, and a third reviewer resolved any discrepancies.

### Eligibility criteria

Studies were eligible for inclusion if they met all of the following criteria:

- Enrolled patients with ICH from one of the major primary etiologies in which surgical timing plays a critical therapeutic role: spontaneous or hypertensive ICH, traumatic ICH, aSAH, or arteriovenous malformation (AVM)-related hemorrhage.
- Evaluated a surgical or procedural intervention aimed at hematoma evacuation or aneurysm securing, including conventional craniotomy, small-bone-window craniotomy, decompressive craniectomy, stereotactic or minimally invasive aspiration, or endovascular coiling/surgical clipping in aSAH.
- Compared at least two clearly defined intervention time windows (e.g., <6 hours, <24 hours, <48 hours, >48 hours, <72 hours, <7 days, <3 weeks).
- Reported clinical outcomes stratified by timing categories, such as Glasgow Outcome Scale (GOS), delayed ischemic neurologic deficit (DIND), rebleeding, intracranial infection, or mortality.
- Were observational in design. Because randomized clinical trials (RCTs) on surgical timing in acute hemorrhage are rarely feasible for ethical and logistical reasons, observational cohorts represent the most informative and clinically relevant evidence base.

Studies were excluded if they:

- Provided only qualitative timing descriptors without numerical definitions.
- Were case reports, case series, reviews, conference abstracts, or otherwise lacked primary comparative data.
- Focused exclusively on secondary causes of ICrH, including venous sinus thrombosis, hemorrhagic transformation, or tumor-related bleeding.
- Evaluated neurosurgical procedures unrelated to the management of primary ICrH.

To enhance comparability and minimize selection bias, we restricted inclusion to the principal etiologies in which surgical timing is directly linked to pathophysiologic progression and treatment urgency. These conditions share a common clinical goal – rapid decompression or prevention of rebleeding – making timing-based comparisons meaningful. We excluded secondary hemorrhage mechanisms (e.g., venous sinus thrombosis, hemorrhagic transformation of ischemic stroke, tumor-associated bleeding), as their indications for intervention, surgical thresholds, and temporal trajectories differ substantially and would confound timing-related analyses.

To ensure transparent and reproducible synthesis, we required explicit quantitative definitions of time windows. Studies that reported only vague or qualitative timing descriptors (e.g., “early” vs. “late” without numerical cutoffs) were excluded because such ambiguity prevents reliable harmonization across datasets.

### Data extraction and definition of outcomes

Two authors independently extracted data utilizing a standardized sheet for data extraction. The subsequent data obtained from each included study: (1) Summary of the studies included and baseline characteristics of the included population; (2) Risk of bias domains; (3) Study outcomes: mortality, rebleeding, DIND, postoperative GOS, and intracranial infection. Disagreements were resolved through consensus.

The GOS was employed to classify patient outcomes, designating scores of 1–3 as unfavorable and 4 or 5 as favorable. Mortality in the included studies was defined as death, deceased status, Modified Rankin Scale (mRS) 6, or GOS 1. We gathered data from multiple time intervals presented in the papers and performed an NMA for comparison. We aimed to ascertain the ideal time frame for surgical intervention to attain the most favorable outcomes.

### Quality assessment

Two authors independently evaluated the quality of each included study. Each study was assessed for quality utilizing the Newcastle–Ottawa Scale (NOS)[24] and the Risk of Bias in Non-randomized Studies of Interventions (ROBINS-I) tool[25]. The NOS assesses studies across three domains: selection, comparability, and outcome. According to the NOS rating system, studies were categorized as poor quality (0–3), moderate quality (4–6), and high quality (7–9). A third author resolved the disputes. The ROBINS-I tool offered a comprehensive evaluation across seven bias domains, categorizing studies into low, moderate, serious, or critical risk of bias. Discrepancies were addressed through consultation with a third author.

### Data synthesis and analysis

A frequentist network meta-analysis was conducted utilizing *P*-scores to rank interventions across all outcomes. Preliminary exploration of SUCRA (Bayesian) values was performed; however, *P*-scores were used for final analyses because of their equivalent ranking interpretation and lower computational complexity. Sensitivity analyses validated the reliability of effect estimates. A random-effects model was employed for data synthesis. The overall effect was demonstrated using an odds ratio (OR) and 95% confidence interval (CI). A *P*-value of less than 0.05 was deemed statistically significant. The Cochrane Q test and the I^2^ test were used to measure heterogeneity. A threshold of *P* < 0.10 or I^2^ > 50% meant a lot of heterogeneity. Sensitivity analyses were conducted by excluding one study at a time and calculating the overall effect of the remaining studies to assess the robustness of the results.

We performed separate pairwise meta-analyses within each subnetwork, comparing surgical timing across predefined windows (<24 vs. >24 hours, < 48 vs. >48 hours, and <72 vs. >72 hours). Subgroup analyses were further stratified by GOS (favorable vs. unfavorable) and by ICH etiology – spontaneous, hypertensive, traumatic, or aneurysmal – selected a priori as the most clinically and pathophysiological relevant factors for surgical timing decisions. Although surgical techniques and comorbidities may act as confounders, inconsistent reporting precluded systematic analysis. Sensitivity analyses assessed the robustness of results, and publication bias was evaluated using Begg’s and Egger’s tests (*P* < 0.05 indicating significance). All analyses were conducted in R software.

We extracted and analyzed the timing categories for surgical intervention exactly as defined in the primary studies. The definitions of “ultra-early,” “early,” and “delayed” surgery varied substantially across studies – for example, some defined ultra-early as within 6 hours, while others defined early as within 24 hours, and delayed intervention thresholds ranged from >24 hours to several days. To avoid introducing bias through arbitrary reclassification, we retained these original definitions and incorporated all reported time windows into the network meta-analysis as provided. This approach ensured that our findings reflect real-world clinical variability rather than standardized cut-offs imposed *post hoc*.

## Results

### Literature search and characteristics of the included studies

**Figure 1.**
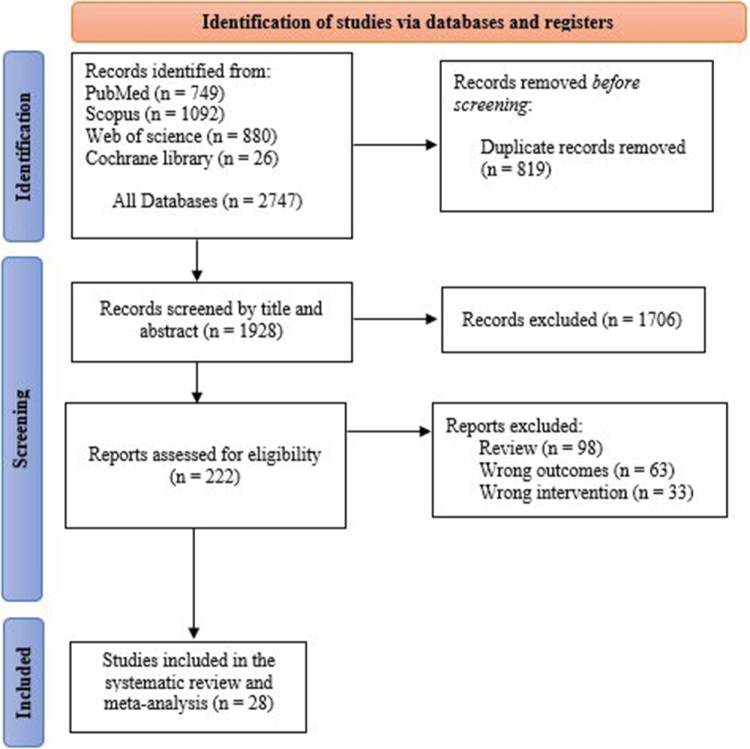
PRISMA flow diagram of studies’ screening and selection.

A thorough literature search resulted in 2747 records. After eliminating 819 duplicate records, 1928 unique articles were screened for title and abstract. After the initial screening, 1706 articles were excluded due to irrelevance. Two hundred twenty-two reports were evaluated for eligibility, excluding 98 review articles, 67 studies with incorrect outcomes, and 33 with unsuitable interventions. A total of 28 studies^[14,16,17,26–50]^, with 5919 patients, satisfied the inclusion criteria and were included in this systematic review and NMA. Figure 1 shows the PRISMA flow diagram of the present study.

The included studies were conducted across various countries, with the highest number from China (9), followed by Italy (2). Single studies were reported from the USA, the UK, Turkey, Brazil, Poland, Belgium, Iran, the Netherlands, New Zealand, Thailand, Egypt, South Korea, and Portugal. The summary of the included studies and patients’ baseline characteristics is presented in Supplemental Digital Content, Table 2, available at: http://links.lww.com/JS9/G455.

According to NOS, the quality assessment of the studies revealed high quality for the observational studies included, except for three with moderate quality. The details of each domain are present in Supplemental Digital Content, Table 3, available at: http://links.lww.com/JS9/G456. The ROBINS-I assessment revealed 20 studies with moderate risk of bias, mainly attributed to residual confounding, and eight studies with serious risk, particularly concerning selection bias and confounding adjustment. The details of the ROBINS-1 assessment are present in Supplemental Digital Content, Table 4, available at: http://links.lww.com/JS9/G457.

### Network meta-analysis

#### Main outcomes

- *Mortality*

**Figure 2.**
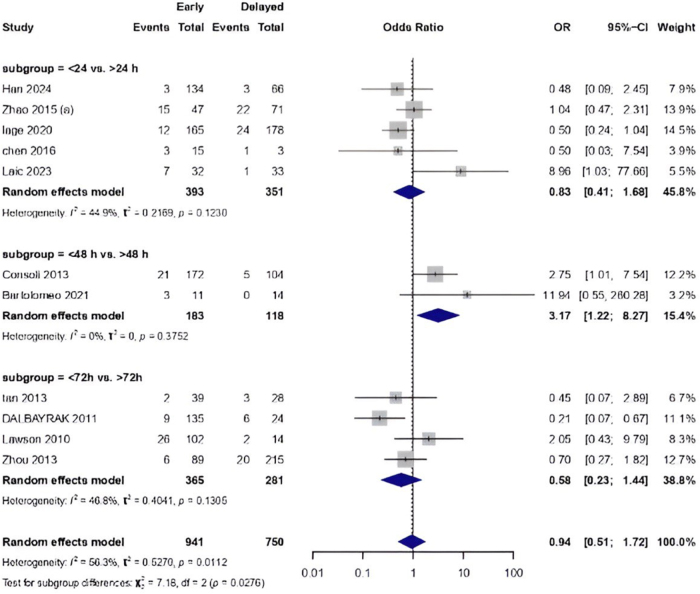
Pairwise meta-analysis comparing early vs. delayed surgery based on mortality rate.

The pairwise meta-analysis indicated a non-significant reduction in mortality for early surgery (within 24 hours) compared to delayed intervention (48 hours and 72 hours) (OR = 0.94, 95% CI: [0.51, 1.72]). Subgroup difference analysis was statistically significant (*P* = 0.0276) with moderate heterogeneity (I^2^ = 56.3%). Mortality outcomes varied across time-based comparisons. For <24 hours versus >24 hours, the OR was 0.83 (95% CI: 0.41, 1.68), showing a nonsignificant reduction. The <48 hours versus > 48 hours subgroup showed a significant increase in mortality risk [OR = 3.17, 95% CI: (1.22, 8.27)]. The <72 hours versus > 72 hours comparison demonstrated a nonsignificant mortality reduction [OR = 0.58, 95% CI: (0.23, 1.44)] (Fig. 2).

**Figure 3.**
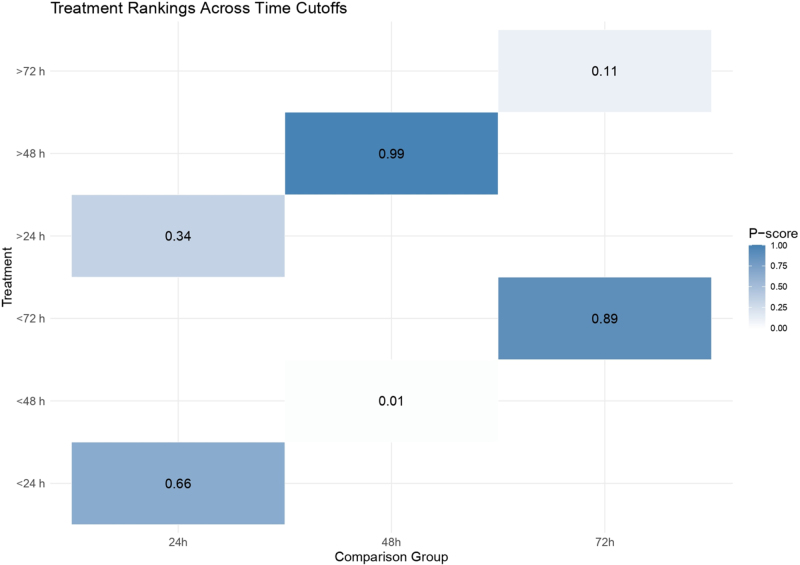
Treatment ranking of mortality risk across surgical timing windows.

Figure 3 shows that the *P*-score ranking indicates the highest probability of reduced mortality for surgery within 48 hours (*P*-score = 0.99), while interventions beyond 72 hours ranked lowest (*P*-score = 0.11).

Subgroup analysis by ICH etiology was statistically significant (*P* = 0.0177). In aneurysmal ICH, surgery within 24 hours did not significantly reduce mortality [OR = 0.70, 95% CI: (0.37, 1.33)], but intervention within 48 hours was associated with a significantly increased risk [OR = 3.17, 95% CI: (1.22, 8.27)]. Surgery within 72 hours did not show a significant benefit [OR = 0.63, 95% CI: (0.34, 1.15)]. In hypertensive ICH, <24 hours surgery showed no significant mortality reduction [OR = 0.48, 95% CI: (0.09, 2.45)]. In traumatic ICH, < 24 hours of surgery was linked with increased mortality [OR = 8.96, 95% CI: (1.03, 77.66)], while in arteriovenous malformations (AVMs), <48 hours [OR = 11.94, 95% CI: (0.55, 260.28)], Supplemental Digital Content, Figure 1, available at: http://links.lww.com/JS9/G453.

A leave-one-out (LOO) sensitivity analysis confirmed stability [OR = 0.94, 95% CI: (0.51, 1.72)], Supplemental Digital Content, Figure 2, available at: http://links.lww.com/JS9/G453. A slightly asymmetric funnel plot suggested a potential small-study effect, Supplemental Digital Content, Figure 3, available at: http://links.lww.com/JS9/G453. Network plot of mortality outcome is shown in Supplemental Digital Content Figure 4, available at: http://links.lww.com/JS9/G453.

- *GOS*

***Favorable GOS (GOS 4 or 5).*** A random-effects analysis of ten studies (*n* = 1003) evaluated early (within 24 hours) versus delayed interventions (48 hours,72 hours, 4–10 days) across three time-interval subgroups. In the 72 hours versus 4–10 days subgroup (2 studies, *n* = 510), no significant difference was found (OR: 0.90, 95% CI: 0.00–202.11), although the estimate exhibited heterogeneity (I^2^ = 48.8%). The subgroup analysis of <72 hours versus >72 hours (3 studies, *n* = 289) indicated a nonsignificant trend favoring early intervention (OR: 0.47, 95% CI: 0.02–13.38), accompanied by considerable heterogeneity (I^2^ = 88.3%). The <24 hours versus >24 hours subgroup (5 studies, *n* = 357) exhibited a nonsignificant trend favoring early intervention (OR: 1.33, 95% CI: 0.32–5.62), accompanied by high heterogeneity (I^2^ = 79.9%). Pooled analysis indicated no significant difference between timing strategies (OR: 0.89, 95% CI: 0.40–1.98), with substantial heterogeneity among studies (I^2^ = 79.6%) and no notable subgroup differences (χ^2^ = 1.25, *P* = 0.53). The findings indicate that although point estimates differ across time frames, no specific intervention timing exhibited statistically significant advantages, Supplemental Digital Content, Figure 5, available at: http://links.lww.com/JS9/G453.

The rankogram indicated significant uncertainty regarding the optimal timing for interventions. Early intervention (<72 hours) demonstrated the highest likelihood of being the optimal strategy (37%); however, its superiority over delayed methods was minimal, exhibiting only a 2%–9% absolute difference in ranking probabilities across various time frames. The lowest-ranked strategy differed among subgroups: for aneurysmal hemorrhages, delayed intervention (>72 hours) was the least effective [OR 1.34 (0.71–2.51)], while for hypertensive/spontaneous hemorrhages, ultra-early intervention (<24 hours) demonstrated the least consistent advantage [OR 3.27 (0.88–12.22)] with a wide confidence interval. No timing strategy exhibited statistically significant superiority overall (*P* > 0.05 for all pairwise comparisons), and all options displayed overlapping uncertainty intervals in the rankogram, indicating clinical equipoise. The consistency of these findings across etiology subgroups (I^2^ = 79.6%) demonstrates that patient-specific factors may be more significant than absolute timing thresholds in clinical decision-making, Supplemental Digital Content, Figure 6, available at: http://links.lww.com/JS9/G453.

Subgroup analysis by ICH cause showed an overall random-effects model OR of 1.21 (0.66, 1.93) with significant heterogeneity (I^2^ = 72%). In aneurysmal ICH, comparing <72 hours to 4–10 days, the OR was 0.90 (0.39, 2.08) (I^2^ = 48.8%), suggesting no significant benefit. Comparing <72 hours versus >72 hours gave an OR of 0.39 (0.03, 4.73) with high heterogeneity (I^2^ = 94.2%), while <24 hours versus >24 hours showed an OR of 2.98 (0.34, 25.82). In hypertensive ICH, <24 hours versus >24 hours yielded an OR of 1.93 (1.17, 3.16). In spontaneous ICH, < 24 hours versus >24 hours showed an OR of 2.22 (0.98, 5.04), while <72 hours versus > 72 hours had an OR of 0.70 (0.19, 2.64), Supplemental Digital Content, Figure 7, available at: http://links.lww.com/JS9/G453.

The LOO sensitivity analysis revealed strong pooled estimates, with the exclusion of any individual study resulting in ORs ranging from 1.01 to 1.43 and 95% confidence intervals that included 1.0. The heterogeneity was moderate to high across the studies (I^2^: 11%–75%, Tau[2] 0.02–0.72), suggesting that no individual study had a disproportionate impact on the overall effect (Supplemental Digital Content, Figure 8, available at: http://links.lww.com/JS9/G453). A network plot of a favorable GOS outcome is shown in Supplemental Digital Content, Figure 9, available at: http://links.lww.com/JS9/G453. Net league of favorable GOS is presented in Supplemental Digital Content, Table 5, available at: http://links.lww.com/JS9/G458.

##### Unfavorable GOS (GOS 1-3)

**Figure 4.**
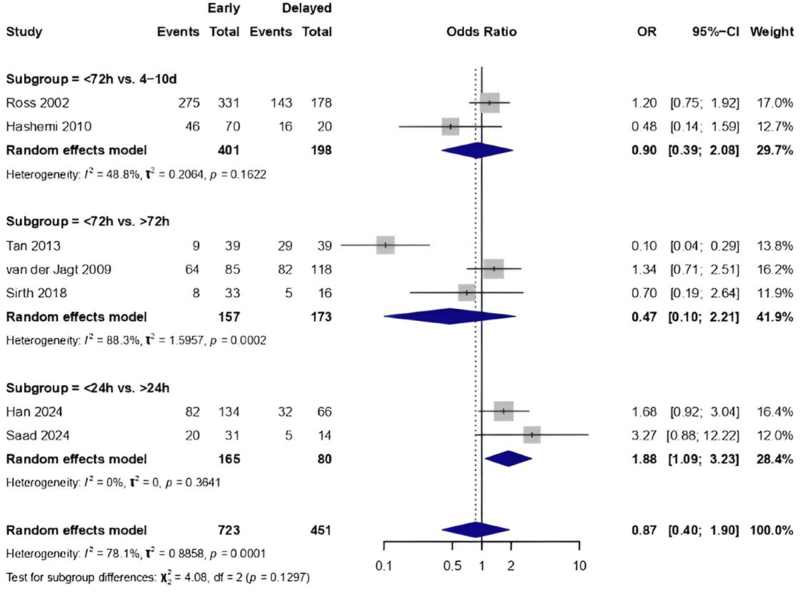
Pairwise meta-analysis comparing early vs. delayed surgery based on unfavorable GOS.

The pairwise meta-analysis found that early surgery within 24 hours significantly reduced the risk of unfavorable GOS compared to later surgery (>24 hours) [OR: 0.50 (0.33–0.76)], with no heterogeneity (I^2^ = 0%). Comparing <72 hours to >72 hours showed no significant difference [OR: 0.89 (0.08–10.05)] and had considerable heterogeneity (I^2^ = 88%). Overall, the pooled OR was 0.54 (0.32–0.92), showing a positive trend favoring early surgery, with moderate variation (I^2^ = 42.6%) (Fig. 4).

The rankogram analysis of unfavorable GOS outcome indicated clear patterns related to the timing of intervention and the etiology of ICH. Most studies indicate nonsignificant trends favoring early treatment for interventions within 24 hours compared to those after 24 hours, particularly in cases involving hypertensive (Han 2024, OR: 0.60; Yuan 2022, OR: 0.41) or spontaneous ICH (Lee 2003, OR: 0.58). The 72-hour subgroup exhibited significant inconsistency; Tan (2013) reported an odds ratio of 0.26, indicating strong support for early intervention in aneurysmal ICH. Sirth (2018) presented an odds ratio of 3.12, suggesting possible harm, Supplemental Digital Content, Figure 10, available at: http://links.lww.com/JS9/G453.

**Figure 5.**
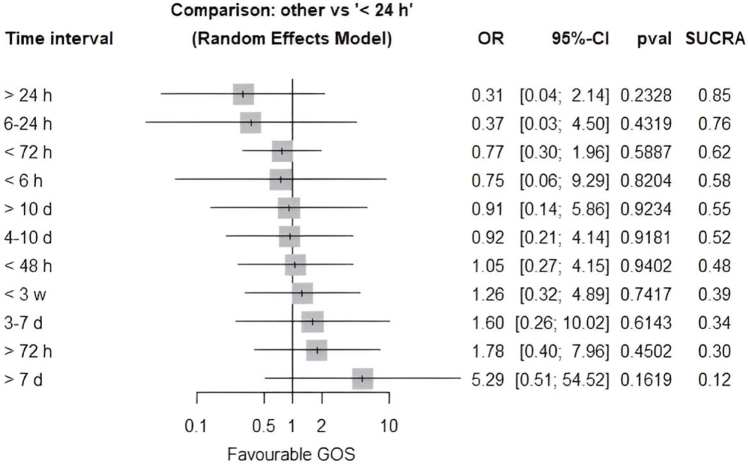
Leave-one-out sensitivity analysis evaluating the robustness of unfavorable GOS results.

The overall random-effects model for etiology subgroups gave an OR of 0.91 (0.40, 2.08), with substantial heterogeneity (I^2^ = 52%). Subgroup difference testing was nonsignificant (*P* = 0.23). For spontaneous ICH, < 24 hours versus >24 hours showed OR 0.63 (0.23–1.69). For aneurysmal ICH, <72 hours versus >72 hours yielded OR 8.53 (0.68–107.11), while <24 hours versus >24 hours yielded OR 0.34 (0.04, 2.91). For hypertensive ICH, <24 hours versus >24 hours gave OR 0.63 (0.23–1.69), Supplemental Digital Content, Figure 11, available at: http://links.lww.com/JS9/G453. The LOO sensitivity analysis assessed the stability of the pooled OR by systematically omitting each study individually. The omission of individual studies resulted in consistent OR estimates, ranging from 0.62 (95% CI: 0.37–1.03) to 1.18 (95% CI: 0.43–3.22), with all confidence intervals crossing 1, indicating nonsignificance, with no heterogeneity detected, I^2^ = 0%, τ 2 = 0, *P* = 0.8869 (Fig. 5). A network plot of unfavorable GOS outcome is shown in Supplemental Digital Content, Figure 12, available at: http://links.lww.com/JS9/G453. The net league of unfavorable GOS is presented in Supplemental Digital Content, Table 6, available at: http://links.lww.com/JS9/G459.

#### Other outcomes

- *DIND*

**Figure 6.**
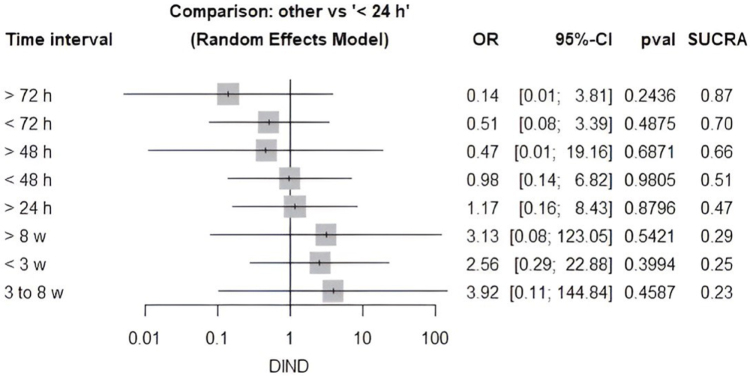
Network meta-analysis comparing different time intervals for surgical intervention and DIND.

An NMA was performed to evaluate the impact of different time intervals on the risk of developing DIND, using <24 hours (early surgery) as the reference category. Delayed interventions conducted beyond 72 hours demonstrated the lowest odds of DIND [OR: 0.14 (0.01–3.81), *P* = 0.2436], whereas those performed within 72 hours exhibited higher odds [OR: 0.51 (0.08–3.39), *P* = 0.4875]. The 48-hour threshold yielded an odds ratio of 0.47 (0.01–19.16) (*P* = 0.6871), suggesting no significant difference. After that, interventions that lasted longer than 8 weeks [OR: 3.13 (0.08–123.05), *P* = 0.5421], less than 3 weeks (OR: 2.56 (0.29–22.88), *P* = 0.3994), and between 3 and 8 weeks [OR: 3.92 (0.11–144.84), *P* = 0.4587] all showed a trend toward higher odds of DIND, but these results were not statistically significant (Fig. 6).

- *Rebleeding*

**Figure 7.**
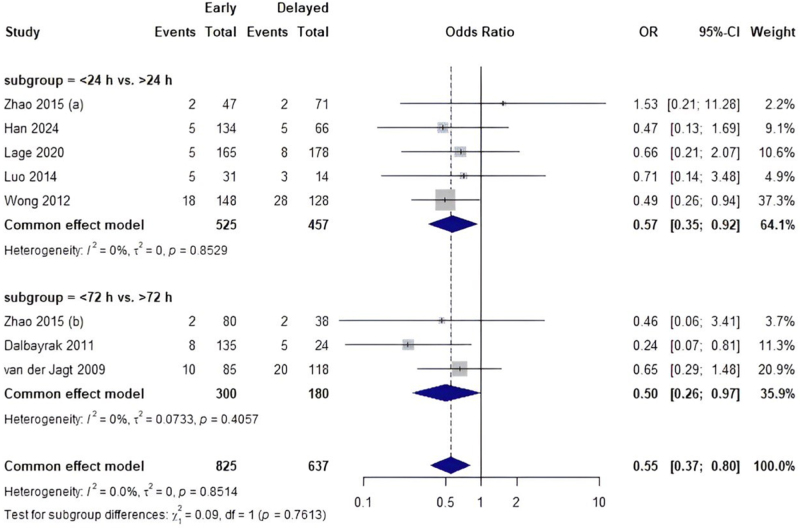
Pairwise meta-analysis comparing early vs. delayed surgery based on rebleeding rate.

A pairwise meta-analysis was performed to assess the effect of early intervention (within 24 hours) compared to delayed intervention (72 hours) on the risk of rebleeding. The results showed that early intervention was linked to a significant drop in the risk of bleeding again [OR: 0.57 (0.39–0.82)], with no variation seen (I^2^ = 0%). Five studies were included in the subgroup analysis of <24 hours versus >24 hours, and they showed that early intervention was linked to a much lower risk of bleeding again [OR: 0.57 (0.35–0.92)]. Four studies were looked at for <72 hours versus >72 hours. They found that early intervention showed no significant difference in decreasing the risk of bleeding again [OR: 0.57 (0.25–1.32)] (Fig. 7).

- *Intracranial infection*

A meta-analysis was performed to evaluate the risk of intracranial infection associated with early (<24 hours) versus delayed (>24 hours) interventions. The pooled estimate indicated no statistically significant difference in the risk of intracranial infection between early and delayed intervention [OR: 0.69 (0.24–2.02)]. No heterogeneity was observed (I^2^ = 0%), Supplemental Digital Content, Figure 13, available at: http://links.lww.com/JS9/G453.

## Discussion

ICH represents a critical neurological emergency necessitating prompt surgical intervention to enhance patient outcomes^[10,12]^. However, the optimal timing of surgery remains a subject of debate, with conflicting evidence regarding its impact on mortality, neurological recovery, and postsurgical complications^[17,36,51,52]^. This systematic review and network meta-analysis aimed to resolve these controversies by assessing the effect of different surgical timing windows on patient outcomes.

Mortality rates and neurological outcomes differ markedly based on the type of ICH, influenced by variations in pathophysiology, anatomical location, and treatment approaches. ICH is frequently linked to elevated early mortality rates and unfavorable long-term functional outcomes, primarily attributable to mass effect, increased intracranial pressure (ICP), and a scarcity of effective medical interventions[53]. SAH, especially when aneurysmal, presents a significant risk of early mortality from rebleeding and delayed cerebral ischemia due to vasospasm, notwithstanding advancements in surgical and endovascular techniques^[5,54]^. In light of these distinct clinical trajectories, we performed a subgroup analysis categorized by the type of ICH to elucidate the impact of the hemorrhage subtype on overall mortality and neurological function. This stratification facilitated a more precise evaluation of prognostic factors and the possible effects of various therapeutic interventions.

Our pooled analysis of 28 studies^[14,16,17,26–50]^ demonstrated that surgical intervention within 48 hours had the highest probability of reducing mortality (*P*-score = 0.99). In contrast, delayed procedures beyond 72 hours were associated with the worst survival probability (*P*-score = 0.11). However, subgroup analyses revealed substantial heterogeneity in benefit according to hemorrhage etiology. In aneurysmal ICH, surgery within 24 hours did not significantly lower mortality, and intervention within 48 hours was associated with a significant increase in mortality risk [OR = 3.17, 95% CI: (1.22–8.27)]. In hypertensive ICH, early intervention within 24 hours showed no statistically significant survival benefit [OR = 0.48 (0.09–2.45)]. For traumatic ICH, ultra-early surgery (<24 hours) was linked to increased mortality (OR = 8.96 [1.03–77.66]). These findings emphasize that the survival benefit of early intervention is not universal and must be weighed against potential risks specific to hemorrhage subtype and patient condition.

Neurological recovery, measured by favorable GOS, did not differ significantly between early and delayed interventions in the overall pooled analysis [OR = 0.89 (0.40–1.98)], and rank probability analysis indicated high uncertainty regarding the optimal timing. Nonetheless, certain trends emerged in etiology-specific subgroups. In hypertensive ICH, surgery within 24 hours was associated with improved odds of favorable recovery [OR = 1.93 (1.17–3.16)], while spontaneous ICH showed a possible advantage for intervention within 24–72 hours. In contrast, aneurysmal and traumatic ICH did not exhibit consistent improvement with ultra-early surgery. For unfavorable GOS, pooled results favored surgery within 24 hours [OR = 0.54 (0.32–0.92)], with the effect most pronounced in hypertensive and spontaneous ICH. These patterns suggest that functional recovery benefits from earlier intervention may be driven largely by nonaneurysmal, nontraumatic subtypes.

Secondary outcomes further highlight the nuanced risk-benefit profile of surgical timing. Early intervention, particularly within 24 hours, was associated with a significantly lower risk of rebleeding [OR = 0.57 (0.35–0.92)], a finding consistent across included studies (I^2^ = 0%). No significant difference in rebleeding risk was observed for the <72 hours versus >72 hours comparison. Importantly, the risk of intracranial infection did not differ between early and delayed interventions [OR = 0.69 (0.24–2.02)], suggesting that modern surgical techniques and infection control measures may mitigate earlier concerns about increased infection rates with prompt surgery. For DIND, interventions beyond 72 hours demonstrated the lowest odds. However, these findings were not statistically significant, indicating that potential vasospasm risk reduction must be balanced against prolonged exposure to the toxic effects of hematoma breakdown products.

When compared with prior literature, our findings refine and, in some cases, challenge earlier conclusions. Meta-analyses by Zhao *et al*[39] and Yao *et al*[18] suggested a general benefit for early surgery but did not clearly define optimal timing or account for etiology-specific risks. The Cochrane review by Whitfield and Kirkpatrick[55] supported early intervention within 3 days but lacked precise timing stratification. Our analysis indicates that while surgery within 48 hours offers the greatest probability of survival benefit overall, this advantage is not consistent across all ICH types. Certain subtypes, such as traumatic ICH, may experience harm with ultra-early surgery, whereas hypertensive and spontaneous ICH show more favorable responses to earlier intervention. These distinctions align with pathophysiological considerations and underscore the etiology-dependent nature of optimal timing. In aneurysmal SAH, particularly after aneurysm securement, surgical evacuation within 24–48 hours may facilitate early relief of mass effect and reduce the risk of secondary ischemic injury. Conversely, in traumatic ICH, ultra-early evacuation (<24 hours) should be approached with caution, as ongoing bleeding, coagulopathy, or associated polytrauma can increase intraoperative and postoperative risks. For hypertensive and spontaneous ICH, earlier intervention, especially within 48 hours, may offer functional recovery benefits, although the survival advantage remains less consistent. These findings reinforce the need to individualize timing decisions based on the underlying cause of hemorrhage, vascular status, and patient stability.

Moreover, our results reinforce the notion supported by Koen *et al*[46] and Zhong *et al*[56] that early intervention significantly improves functional outcomes, with the highest probability of achieving favorable GOS scores observed in patients operated on within 24 hours. However, our analysis further distinguishes the varying benefits of early surgery across different ICH subtypes, revealing that while hypertensive and traumatic ICH may benefit from early intervention, aneurysmal ICH does not uniformly exhibit the same survival benefit, thus suggesting that optimal timing is highly etiology-dependent. This nuanced approach resolves prior controversies by indicating that a one-size-fits-all timing strategy may be inappropriate.

The pathophysiological rationale for these findings is grounded in the dual-phase injury model of ICH. The initial insult produces a mass effect, elevated ICP, and risk of herniation, key predictors of early mortality, while secondary injury mechanisms such as perihematomal edema, oxidative stress, inflammation, and blood–brain barrier disruption evolve over subsequent hours to days. Early intervention (<48 hours) can alleviate mass effect and reduce secondary injury progression; however, this must be balanced against risks such as intraoperative rebleeding in fragile vascular lesions or during unstable hemostatic states. Delayed surgery may allow stabilization and better control of underlying vascular pathology, potentially reducing risks such as DIND, but at the cost of prolonged exposure to hematoma toxicity and increased ICP^[56–60]^.

From a clinical perspective, our findings highlight that surgical timing in ICH must be individualized and guided by hemorrhage etiology, patient stability, and the feasibility of definitive lesion control. For instance, a 24–48-hour intervention may be optimal in selected aneurysmal SAH patients once the aneurysm has been secured. In contrast, ultra-early surgery in traumatic ICH should generally be avoided, unless there is imminent herniation, due to the heightened risk of exacerbating bleeding in the context of coagulopathy or unstable vascular injury. In hypertensive or spontaneous ICH, earlier intervention may support better functional recovery, though survival benefits remain less consistent. Advances in minimally invasive evacuation, endoscopic techniques, and antibiotic-impregnated catheters have further reduced complication risks, enabling a tailored, etiology-specific approach that aims to optimize survival and functional outcomes while minimizing preventable harm^[56–60]^.

The magnitude and nature of benefit from early surgery differ substantially across ICH subtypes, reflecting both underlying pathophysiology and procedural considerations. In hypertensive and spontaneous ICH, early evacuation, particularly within 24–48 hours, may effectively reduce mass effect, limit intracranial pressure elevation, and mitigate secondary injury from perihematomal edema and inflammatory cascades^[56–60]^. These subtypes often lack fragile vascular lesions, lowering the risk of intraoperative rebleeding and allowing earlier intervention to be both safer and more effective^[56–60]^.

In contrast, traumatic ICH presents unique challenges: ultra-early surgery (<24 hours) may be performed before coagulopathy is corrected or concomitant injuries are stabilized, thereby increasing the likelihood of intraoperative hemorrhage expansion or postoperative complications. The dynamic nature of traumatic lesions, with evolving hematomas and cerebral swelling, may further diminish the benefit of immediate evacuation^[56–60]^.

For aneurysmal SAH with associated ICH, the timing of surgery must be carefully aligned with aneurysm securing. Hematoma evacuation before definitive aneurysm obliteration risks precipitating catastrophic rebleeding. Consequently, the survival and functional benefits of early evacuation in this setting are generally realized only after the aneurysm has been treated, with an optimal window likely within 24–48 hours thereafter^[56–60]^.

These distinctions emphasize that early surgery is not uniformly advantageous across all ICH etiologies. The decision must balance the urgency of mass effect relief against the procedural risks specific to the underlying pathology, vascular status, and systemic condition of the patient.

### Clinical implications

This analysis offers a decision framework driven by etiology, which is of principal clinical value. This advocates for postponing surgery in traumatic ICH until stabilization, emphasizing the importance of 24–48-hour evacuation in secured aSAH, and recommending earlier intervention (within 48 hours) in hypertensive or spontaneous ICH to optimize functional recovery – thus tailoring surgical timing according to the specific pathology involved.

### Future perspectives

Focused research on hemorrhage etiology is needed to improve surgical scheduling in ICH. Future studies should prioritize prospective, multicenter trials that identify patients by ICH subtype, hypertensive, aneurysmal, and traumatic, to better assess early versus delayed therapies. Hybrid treatments such as endovascular aneurysmal ICH with phased hematoma evacuation or optimal coagulopathy management for trauma-related ICH should be prioritized^[15,57–59]^.

Advanced imaging modalities like CT perfusion, susceptibility-weighted MRI, and emerging biomarkers like GFAP and UCH-L1 may improve patient classification. The nonsignificant tendency toward reduced DIND with delayed surgery requires more extensive cohort studies to ascertain if this benefit is due to ischemia risk reduction or survivor bias^[48–50]^.

The apparent lack of infection rate variation across surgical timings warrants further study in various clinical settings to determine if modern aseptic techniques, such as antibiotic-impregnated devices and intraoperative antimicrobial irrigation, consistently reduce historical infection. Artificial intelligence might also combine age, comorbidities, and hematoma features to personalize timing decisions. Establishing solid, evidence-based standards requires global registries to record long-term outcomes beyond mortality and GOS, including cognitive and quality-of-life indicators^[45–50]^.

Although early surgery did not decrease mortality rates, its functional advantages indicate that timing decisions ought to be customized to the specific profiles of individual patients. Considerations include baseline prognosis, surgical accessibility, and care objectives (e.g., survival versus functional independence). Frameworks for shared decision-making are essential for incorporating these outcomes into clinical practice.

Additionally, the analysis of adjunctive procedures, including ICP monitoring and external ventricular drainage (EVD), was not feasible due to variability in reporting among the studies. The interventions are expected to influence decisions regarding surgical timing, especially in scenarios involving hydrocephalus or considerable mass effect. Future studies must standardize the reporting of these essential management details to enhance the understanding of their synergistic effects in relation to surgical timing.

Furthermore, the use of artificial intelligence and machine learning techniques on extensive, multicenter datasets offers potential for optimizing surgical timing tailored to individual patients. These models have the potential to incorporate complex variables such as imaging characteristics, genetic markers, and real-time physiological data to produce personalized predictions regarding optimal surgical timing, thereby advancing beyond the general categories examined in this study.

### Strengths and limitations of the study

This study’s strengths include its systematic review and network meta-analysis methodology, which thoroughly appraise surgical timing across various ICH subtypes. The etiology-specific subgroup analyses yield significant insights into disparate treatment responses, augmenting clinical relevance. Standardized outcome measures and a solid analytical framework further enhance methodological rigor.

This study’s primary advantage compared to previous meta-analyses lies in its use of a network meta-analysis framework alongside predefined, etiology-focused subgroup analyses. This approach facilitates the concurrent comparison of various surgical time windows and, importantly, reveals that the advantages of early surgery are not consistent, but rather significantly influenced by the underlying etiology of hemorrhage, a detail overlooked by previous studies that regarded ICH as a uniform condition.

However, several limitations must be acknowledged. Most included studies were retrospective with heterogeneous designs, introducing potential bias and limiting causal inference. Variability in patient selection, surgical techniques, and postoperative management likely contributed to inconsistencies in outcomes. The broad and sometimes overlapping timing classifications (e.g., <24 hours nested within <48 hours) may have diluted effect sizes and obscured timing-specific differences. Although we retained all reported timing categories to reflect real-world practice and preserve external validity, this approach may reduce the precision of timing-related estimates. Future meta-analyses would benefit from adopting standardized definitions or performing sensitivity analyses that exclude studies with nonstandard or overlapping categories.

The strength of evidence for some small subgroups, particularly ultra-early surgery in traumatic ICH (*n* = 65), remains limited, making these findings more susceptible to random error and study-level bias; they should therefore be interpreted with caution. High statistical heterogeneity was also observed in certain analyses, such as GOS with <24 hours of intervention, likely arising from differences in baseline severity, patient selection, and surgical expertise, which may limit the precision of pooled estimates.

In addition, our analysis encompassed studies involving high-risk ICH, characterized by poor Hunt-Hess/WFNS grades and large hematomas; however, data regarding patients with fixed or dilated pupils or irreversible herniation were limited. The cohorts analyzed predominantly included salvageable cases, with moribund patients frequently excluded from consideration. In this subset, ultra-early surgery may not enhance outcomes, as demonstrated in poor-grade aSAH[45]. At the same time, delayed intervention poses the risk of missing the therapeutic window for reversible deficits. Future research should standardize the reporting of clinical severity markers, such as pupillary reflexes and intracranial pressure, to enhance timing protocols for catastrophic ICH.

Moreover, this analysis is limited by the variability in definitions of timing windows across primary studies (e.g., “early,” “ultra-early”). Our objective was to determine the optimal timing for intervention; however, we faced limitations due to the differing thresholds established by the protocols of the included studies. In response to this issue, we analyzed all reported time windows without applying arbitrary standardization, thereby ensuring that our conclusions accurately represent real-world clinical variability. Future studies should implement standardized definitions to facilitate more accurate comparisons.

Furthermore, the absence of reliable data on the use of intracranial pressure monitoring and concurrent operations such as ventricular draining precluded an examination of their potential confounding effects or synergistic roles with surgical timing.

## Conclusion

Our NMA provided significant insights into the optimal timing for surgical intervention in ICH. It indicates that early surgical procedures conducted within 24–48 hours yield the most tremendous benefits in terms of mortality reduction. Nevertheless, the findings exhibited variability in aneurysmal ICrH, as ultra-early surgical intervention did not present survival advantages. It is worth emphasizing the necessity of incorporating the etiology of the hemorrhage when establishing surgical timing, given that the benefits of early intervention differ among various causes of ICH. While advancements in surgical methodologies and infection management have alleviated some prior concerns, our findings stress the imperative for personalized treatment approaches. Further validation through prospective trials is crucial to refining timing protocols and improving patient outcomes.

## Data Availability

On reasonable request, the supporting data for this study’s findings can be provided by the corresponding author.

## Research registration unique identifying number (UIN)

The PROSPERO registration number for this systematic review and meta-analysis is CRD420251017366.
